# Glucocorticoids in Sepsis: To Be or Not to Be

**DOI:** 10.3389/fimmu.2020.01318

**Published:** 2020-07-21

**Authors:** Jolien Vandewalle, Claude Libert

**Affiliations:** ^1^Center for Inflammation Research, VIB, Ghent, Belgium; ^2^Department of Biomedical Molecular Biology, Ghent University, Ghent, Belgium

**Keywords:** sepsis, glucocorticoids, HPA axis dysfunction, HAT therapy, glucocorticoid resistance

## Abstract

Sepsis is a highly lethal syndrome resulting from dysregulated immune and metabolic responses to infection, thereby compromising host homeostasis. Activation of the hypothalamic–pituitary–adrenal (HPA) axis and subsequently adrenocortical glucocorticoid (GC) production during sepsis are important regulatory processes to maintain homeostasis. Multiple preclinical studies have proven the pivotal role of endogenous GCs in tolerance against sepsis by counteracting several of the sepsis characteristics, such as excessive inflammation, vascular defects, and hypoglycemia. Sepsis is however often complicated by dysfunction of the HPA axis, resulting from critical-illness-related corticosteroid insufficiency (CIRCI) and GC resistance. Therefore, GCs have been tested as an adjunctive therapy in sepsis and septic shock in different randomized clinical trials (RCTs). Nonetheless, these studies produced conflicting results. Interestingly, adding vitamin C and thiamin to GC therapy enhances the effects of GCs, probably by reducing GC resistance, and this results in an impressive reduction in sepsis mortality as was shown in two recent preliminary retrospective before–after studies. Multiple RCTs are currently underway to validate this new combination therapy in sepsis.

## The Sepsis Burden

Sepsis is defined as a life-threatening organ dysfunction due to a dysregulated host response to an infection (1). In 2017, the World Health Organization (WHO) has labeled sepsis as the most urgent unmet medical need of our times (2). Despite that the term “sepsis” was already introduced by Hippocrates in the fourth century BC and innumerable patients worldwide have died of it, sepsis is not well-known in the general population (2). A recent study has proven that the incidence of sepsis was even severely underestimated and is at least twice of what was previously thought (3). The annual burden of sepsis is now estimated to be 48.9 million sepsis cases with 11 million deaths worldwide, representing 19.7% of all global deaths (3). The previous estimates of sepsis incidence and mortality relied on hospital administrative databases, excluding patients who were never admitted to hospitals, e.g., in developing countries. Moreover, the old estimates were based on middle- and high-income countries, and many studies were restricted to adults as a result of a paucity of data for children (3).

During the beginning of the twenty-first century, the frequency of sepsis has increased, while mortality rates decreased. The increasing frequency reflects the increasing aging population who has impaired immunity as a result of immunosenescence, whereas the sepsis mortality decrease is thought to be a consequence of improved health care in hospitals and ICUs (4). Unfortunately, the decreased mortality rates are not a consequence of new therapies or a better understanding of sepsis pathobiology based on preclinical studies. This failure in progress of sepsis research is not a result of a lack of studies. On the contrary, sepsis research in mice has yielded many new potential therapies. However, none of these strategies have successfully penetrated to the bedside (5). Therefore, management of sepsis patients is supportive rather than curative and essentially relies on antibiotic treatment, hemodynamic stabilization, and support of failing organs, e.g., ventilation of the lungs. Next to the obvious global health problem, sepsis also poses a high economic burden. The mean hospital-wide cost of a sepsis patient is estimated to be $ 32.421 (6). Moreover, patients who survive sepsis often have long-term cognitive impairment and functional disability with a permanent high risk of mortality after discharge (7).

Sepsis is caused by an infection, and the primary site of infection is most commonly the respiratory tract (64%), followed by the abdomen (20%) and urogenital tract (14%). Intra-abdominal infection is associated with the highest mortality (30.7%) (8). In most cases, sepsis is caused by gram-positive or gram-negative bacteria, but also fungi, viruses, and parasites can cause sepsis. Preclinical models of sepsis can be classified into three categories based on the mechanism involved, including administration of a cytokine or toxin [e.g., tumor necrosis factor (TNF) or lipopolysaccharide (LPS)], administration of a viable pathogen (e.g., *E. coli*), or disruption in the animal's protective barrier allowing for bacterial invasion [e.g., cecal ligation and puncture (CLP)] (9). The latter is considered as the gold standard for sepsis research (10). Nevertheless, in the current animal models of sepsis, most deaths occur within the first 5 days, thereby representing only the early deaths of sepsis resulting from the initial hyper-inflammatory state. In many cases, sepsis patients can survive the initial hyper-inflammatory state and succumb from subsequent nosocomial infections with pneumonia being the most common etiology (11, 12). Indeed, as the septic condition persists, the host immunologic response shifts from a hyper-inflammatory state to anti-inflammatory in which the patient is immunocompromised. A preclinical model studying late-onset immunosuppression in sepsis is the “two-hit model” of sepsis in which a primary sublethal infection, such as CLP, impairs the immune system, thereby rendering the host more susceptible to secondary infections, such as pneumonia (12, 13).

## HPA Axis and GR

The immune system protects the host against infections by eliminating the infectious agents (i.e., resistance mechanism). However, pathogen eradication is frequently associated with collateral tissue damage and inflammation, which potentially decreases host fitness (14). Activation of the hypothalamic–pituitary–adrenal (HPA) axis by immune cell-derived cytokines is an important regulatory process to maintain homeostasis and survive the life-threatening impact of excessive inflammation on the host (i.e., tolerance mechanism) (14).

**Figure 1:**
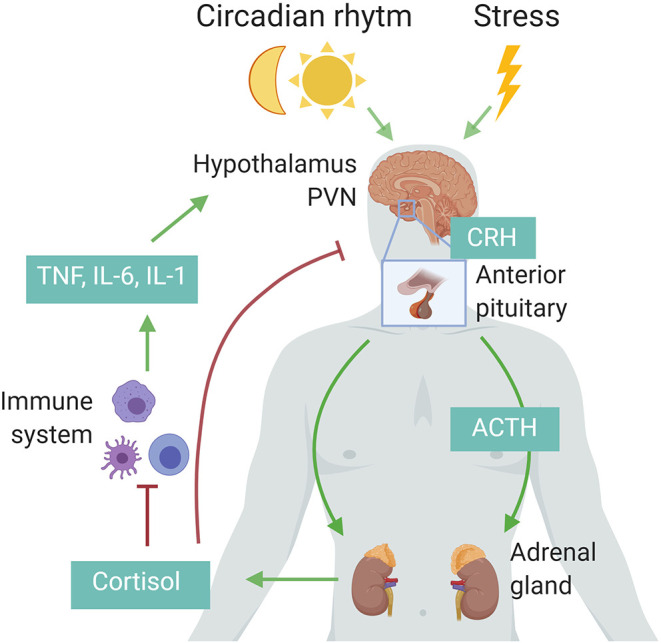
Schematic representation of the hypothalamic–pituitary–adrenal (HPA) axis. Upon circadian rhythm, stress, and inflammatory cytokines, the hypothalamus secretes corticotropin-releasing hormone (CRH), stimulating the pituitary to release adrenocorticotropic hormone (ACTH), which in turn induces cortisol (human) or corticosterone (rodents) by the adrenal cortex. These glucocorticoids in turn negatively regulate the activity of the HPA axis via the paraventricular nucleus (PVN) of the hypothalamus and the anterior pituitary, or indirectly by decreasing the expression of inflammatory cytokines. Figure created with BioRender.com.

The HPA axis is regulated by a circadian and ultradian rhythm characterized by peak levels during the active phase which is in the morning in humans and in the beginning of nighttime in nocturnal animals such as mice. The activity of the HPA axis is further increased upon physiological (e.g., activated immune system) and emotional stress (15). Cytokines such as interleukin 1β (IL-1β), TNF, and IL-6 are potent inducers of the HPA axis (Figure 1) (16). The hypothalamus secretes corticotropin-releasing hormone (CRH) which subsequently induces secretion of adrenocorticotropic hormone (ACTH) by the anterior pituitary and finally glucocorticoids (GCs; cortisol in humans and corticosterone in rodents) from the adrenal cortex using cholesterol as a substrate. The lipophilic GCs are then released into the bloodstream, diffuse through cell membranes to bind cytosolic glucocorticoid receptor (GR), which is constitutively and ubiquitously expressed throughout the body. Fine-tuning of blood GC levels is regulated by a negative feedback loop, whereby increased GC levels inhibit the HPA axis at different points (17). Adrenal GC production controls total GC levels in the circulation, but extracellular binding proteins and intracellular enzymes regulate GC activity locally. In plasma, a large proportion of circulating GC is bound with corticosteroid-binding globulin (CBG) or albumin, thereby leaving only ~5% of circulating GC in a free, biological active form. At inflammatory sites, neutrophil elastases can cleave CBG, thereby liberating free GC at the site of inflammation. Within cells, 11β-hydroxysteroid dehydrogenase (11β-HSD1/2) enzymes regulate the interconversion of bioactive cortisol/corticosterone and inactive cortisone/11-dehydrocortisone. Inflammatory cytokines can regulate the expression of the 11β-HSD enzymes, thereby modulating GC activity locally (18).

In the absence of ligand, the GR is primarily located in the cytoplasm in a large chaperone complex. Upon ligand binding, GR undergoes a conformational change leading to partial dissociation from the chaperone complex exposing its two nuclear localization signals (NLS). This leads to nuclear translocation (NTL) of the GR, where it can interact with other proteins and DNA to influence gene expression (19). GR regulates gene expression of up to 20% of the genome by working as a monomer or as a homodimer (18). In mouse liver under endogenous GC levels, GR binds to DNA more frequently as a monomer than as a dimer. As a monomer, GR interacts with DNA by binding to GC response element (GRE) half-sites. If a binding site for another transcription factor (TF) is nearby the half site, both elements may act as a composite site where there is an interaction (positive or negative) between the GR monomer and the other TF (20). Alternatively, two GR monomers can also interact with DNA by binding to inverted negative GREs (IR-nGREs) to specifically repress gene transcription upon GR binding by recruiting corepressors and histone deacetylases (21). Administration of supra-physiological GC doses in contrast favors GR dimer binding at GREs at the cost of monomer binding (22). Binding of GR homodimers to GRE sequences leads to an enhancement of gene expression (also referred to as transactivation) (22). These data indicate that GR monomers are more important for the physiological functions of GR, whereas GR dimers are important for their pharmacological and perhaps stress functions (22). GR can also occupy specific genome regions indirectly in a mechanism known as tethering. Tethering involves physical interaction of the monomeric GR with another TF, such as activator protein 1 (AP-1) and NF-κB (23). These interactions influence the DNA binding, cofactor recruitment, and gene transcription of the involving TF. Besides the genomic effects, GCs also exert rapid non-genomic effects that do not require transcription processes or protein synthesis (24).

## Role of Endogenous GCs in Sepsis

To establish whether activation of the HPA axis is essential for survival of sepsis, several animal studies were performed. Disrupting the HPA axis surgically (by removal of either the pituitary gland or adrenal cortex) or pharmacologically (with the GR antagonist RU486) sensitizes mice for sepsis (25–27). These data are in accordance with human studies showing that patients with Addison's disease, i.e., showing primary adrenal insufficiency and hypocortisolism, have an increased risk of death following infections (28). In contrast, overexpression of GR leads to reduced inflammatory responses and increased survival following endotoxic shock (29). SPRET/Ei mice, which are known for their resistance for endotoxic shock, display a combination of increased GR levels and overactivation of their HPA axis (30), and this is associated with higher levels of the GR inducible gene *Tsc22d3* encoding the GC-induced leucine zipper (GILZ) protein (31). RU486 pretreatment or adrenalectomy abolishes the resistant phenotype of SPRET/Ei mice.

**Figure 2:**
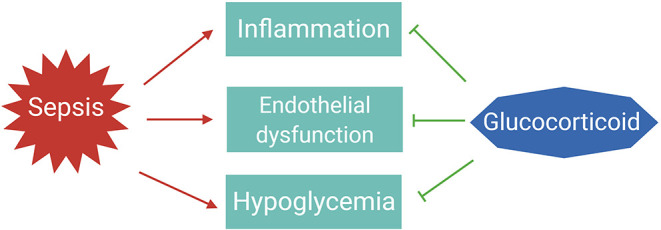
The essential roles of glucocorticoids to tolerate sepsis. In sepsis patients, the immune system is activated to eliminate the infectious agents; however, if not properly balanced, pathogen eradication is associated with excessive inflammation and endothelial barrier dysfunction leading to organ failure. Dysregulated immune responses are also accompanied with changes in metabolism, such as hypoglycemia. Glucocorticoids are induced upon infections to maintain homeostasis and tolerate the life-threatening impact of sepsis on the host.

The essential role for GCs in survival of sepsis is thought to be the result of their anti-inflammatory, vascular, and gluconeogenic effects (Figure 2) (32).

### Effect of GR Signaling on Inflammation

Activation of the HPA axis is clearly important in the protection against inflammation. As the GR is an ubiquitously expressed ligand-dependent transcription factor, it can regulate many different gene networks, each uniquely determined by particular cellular and physiological contexts (33). To precisely dissect mechanisms involved in a physiological process, cell-specific GR deletion may render information on its role in a particular cell type. Indeed, cell- or tissue-specific deletion or inhibition of the GR has demonstrated the crucial role for GR in dampening the inflammatory response during sepsis in different cells or tissues (see Table 1).

**Table 1:** Implications of cell-specific GR deletion on sepsis progression.

**Tissue/cell**	**GR deletion**	**Effect of GR deletion in sepsis model**	**References**
Liver	*shRNA-GR*	Excessive liver and systemic inflammation Increased mortality (CLP)	(34)
IEC	*Villin cre*	Increased ISG expression in gut, intestinal permeability Increased mortality (LPS, TNF)	(35, 36)
Skeletal and cardiac muscle	*Mck cre*	Reduced muscle atrophy (LPS)	(37)
Vascular smooth muscle	*SM22-α cre*	Small trend toward increased mortality (LPS)	(38)
Endothelial cell	*Tie-1 cre*	Hemodynamic instability, higher NO levels, excessive systemic inflammation Increased mortality (LPS)	(38)
Myeloid cells/macrophages	*LysM cre*	Excessive systemic inflammation, ALI Increased mortality (LPS)	(36, 39–41)
Thymocytes/T-cells	*Lck cre*	Resistance to CLP-induced thymocyte apoptosis Increased mortality (CLP)	(42)
DCs	*CD11c cre*	Excessive systemic inflammation (IL-12) Increased mortality (LPS)	(43)
NK cells	*Ncr1 cre*	Increased IFN-γ production, spleen immunopathology Increased mortality (CMV, LPS)	(44, 45)

*IEC, intestinal epithelial cell; CLP, cecal ligation and puncture; LPS, lipopolysaccharide; TNF, tumor necrosis factor; ISG, interferon-stimulated genes; NO, nitric oxide; ALI, acute lung injury; DC, dendritic cell; NK, natural killer; CMV, cytomegalovirus*.

In the past, the immune suppressive effects of GCs were believed to be the result of monomeric GR-mediated tethering to key inflammatory transcription factors such as NF-κB and AP-1. However, the pivotal role of GR-mediated transrepression as a mechanism to limit inflammation has been challenged by several studies using dexamethasone as a synthetic ligand for the GR. By inhibiting *de novo* protein synthesis by use of cycloheximide, dexamethasone-dependent repression of multiple mRNAs (IL-8, CSF2, CXCL1) was blocked. This implies repressive mechanisms involving gene transcription. Conversely, other pro-inflammatory mRNAs (e.g., TNF, ICAM1) showed dexamethasone-dependent repression that was unaffected by cycloheximide and thus implies repressive mechanisms involving classical transrepression (46). The pro-inflammatory mRNAs showing attenuated repression by dexamethasone in the presence of cycloheximide were significantly more repressed and were more potently repressed compared to those mRNAs showing cycloheximide-independent repression, thereby indicating the primacy of GR-mediated transactivation as a predominantly anti-inflammatory mechanism. *In vivo* evidence supporting a role for GR-mediated transactivation in mediating the anti-inflammatory effects of GCs was provided by using GR^dim/dim^ mice characterized by a defective GR dimerization yet an intact monomer profile (47). Several studies showed that GR homodimers are indispensable for the GC-mediated protection in acute inflammation. Indeed, GR^dim/dim^ mice are highly susceptible for TNF-induced mortality as these mice fail to induce the *Dusp1* gene, coding for MAPK phosphatase 1 (MKP1) (48). Moreover, GR^dim/dim^ mice display poor control of intestinal STAT1 resulting in excessive necroptosis in the gut after TNF injection (35). GR dimerization is also required for survival against LPS- and CLP-induced shock via downregulation of *Il-1*β (41) and upregulation of *SphK1* (40) in macrophages. GR^dim/dim^ mice present impaired lung function upon LPS challenge under intensive care treatment, possibly through regulation of Osteopontin (Opn) in lung tissue (49). So, GR dimers have the potential to control acute inflammation because they induce dimer-dependent genes, the product of which has anti-inflammatory functions (e.g., SPHK1, MKP1) or by repressing pro-inflammatory genes (e.g., *Stat1, Il-1*β) in a dimer-dependent way.

Some researchers feel it counterintuitive to use immune suppressive drugs, such as GCs, in patients with severe infections. However, in spite of the well-known anti-inflammatory actions of GCs, it is becoming increasingly clear that GCs also display immune-enhancing effects in sepsis. A better comprehension of the impact of GCs on the immunological effects may provide rationale for their use in sepsis.

#### Myeloid Cells

Myeloid-specific deletion of GR leads to an increased susceptibility to LPS-induced mortality (39–41). In contrast, targeting dexamethasone specifically to macrophages by use of an anti-CD163-dexamethasone conjugate increases the anti-inflammatory potential of dexamethasone resulting in enhanced protection against LPS (50). The essential functions of myeloid GR in protecting against LPS-induced mortality is to inhibit pro-inflammatory mediators, such as IL-1β (41) and p38 MAPK (39), as well as to induce the expression of anti-inflammatory genes, such as *SphK1* (40). In addition to the well-known anti-inflammatory actions of GR, GCs have been described to enhance the phagocytic and bacterial killing capacity of monocytes/macrophages, which is off course of high value in a bacterial environment like sepsis. *In vitro*, incubation of human monocytes with dexamethasone augments the phagocytosis of various particles, such as *Staphylococcus aureus*, latex beads, zymosan, acLDL and myelin, and this effect could be completely blocked by adding the GR antagonist RU486 during culture (51). Not only the phagocytosis of *S. aureus* bacteria is augmented but also the killing of these bacteria is increased by dexamethasone (51). Equally so, peripheral blood monocytes derived from sepsis patients who were treated with hydrocortisone *in vivo* display enhanced phagocytosis in an *ex vivo* assay, whereas the pro- and anti-inflammatory responses are attenuated in these patients (52). Also *in vivo*, there is evidence for the immune-enhancing effects of GCs. Administration of low-dose GCs in mice challenged with *E. coli* decreases the bacterial burden of these mice (53). Overexpression of the GR-inducible protein GILZ in the whole body (54) or restricted specifically to macrophages (55) protects mice against CLP-induced sepsis by limiting systemic inflammation, while bacterial clearance is increased in these mice. It would be interesting to study whether GR^Lysmcre^ mice are also sensitized in bacterial sepsis models such as the CLP model and, if so, whether this could be linked to an increased bacterial burden along with increased inflammation. Next to enhancing phagocytosis of bacteria, GCs also enhance the clearance of apoptotic cells and cell debris, i.e., efferocytosis, which is important to start tissue repair [see recent review on this topic; (56)]. A possible mechanism explaining the apparently opposing effects of GCs in sepsis is by a GC-induced differentiation of a specific anti-inflammatory subtype of monocytes, which seems to be actively involved in resolution of inflammatory reactions instead of a global suppression of monocytic effector functions as was originally thought (57).

#### T-Cells

Using GR^Lckcre^ mice in whom thymocytes lack the GR, it has been demonstrated that GR signaling is required for proper selection of thymocytes and absence of GR signaling in thymocytes impairs thymocyte development resulting in generation of functionally compromised T-cells (58). The resulting immunocompromised state renders these mutant mice more susceptible for CLP-induced sepsis (42). In the latter study, only survival curves are provided, but neither the inflammation state nor bacterial burden of the septic animals is shown. Further evidence for the role of GCs in enhancing T-cell responses was recently provided by Shimba et al. They demonstrate that endogenous GCs drive IL7-R expression in a diurnal fashion which induces T-cell homing to peripheral lymphoid organs via CXCR4 expression, thereby enhancing the adaptive immune response against *Listeria monocytogenes* infection (59).

#### Dendritic Cells (DCs)

To explore the function of GR on DCs in sepsis, mice with selective deletion of the GR in DCs (GR^CD11ccre^) were subjected to LPS injection (43). These mutant mice are much more susceptible to LPS-induced shock as GR on DCs is important to reduce IL-12 production, a cytokine that causes secretion of other inflammatory mediates (43), probably by skewing DC maturation toward a tolerogenic profile by inducing GILZ (60). In addition, endogenous GCs produced upon LPS stimulus causes the death of a subset of DCs that are the primary producers of IL-12, thereby inducing LPS tolerance (43). As DC cell loss is a hallmark for sepsis-induced immune dysfunction, its suppression by GCs may contribute to post-septic immunosuppression, especially given the essential role for DCs in antigen presentation. Therefore, it would be informative to test these mutant mice in a bacterial model of sepsis and check whether bacterial clearance is affected.

#### Natural Killer Cells (NK Cell)

Deletion of GR specifically on NRC1+ cells (i.e., NK cells and type I innate lymphoid cells) renders mice more susceptible to both mouse cytomegalovirus (CMV) (44) and LPS-induced mortality (45). The endogenous GCs produced in response to infection protect by inducing PD-1 on NK cells, thereby controlling the production of IFN-γ and leading to tolerance in both infectious conditions (44). Interestingly, this GC-PD1 pathway only limits the production of IFN-γ, but viral clearance is not compromised in these mice (44). These data suggest that GCs are able to decouple the IFN-γ-producing function of NK cells from their cytotoxic function. The mechanism underlying this decoupling effect of GCs needs further investigation. It would be interesting to evaluate the role of this pathway in other infectious conditions as NK cells are also known to be involved in the host immune response against bacterial and fungal pathogens. Endogenous GCs are also important for NK cell function in patients with respiratory infections. Patients with Addison's disease display a significantly decreased NK cell cytotoxicity, thereby compromising early recognition and elimination of virally infected cells. This impaired antiviral immune defense may contribute to the increased rate of respiratory infections and death typically observed in these patients (28).

In contrast to the aforementioned studies, other studies show an impairment of phagocytosis or pathogen clearance upon GC addition (61–64). Possible explanations for this discrepancy include the dose and type of GC used, duration of treatment, the activation state and origin of the cells (*in vitro* differentiated or obtained from tissues), and the phagocytosis assay used.

To conclude, the abovementioned studies clearly demonstrate a role for GCs in dampening the exaggerated immune response, while at the same time pathogen clearance can be preserved. Nevertheless, further studies are warranted to fully understand the mechanism and consequences of GCs in sepsis and to predict the risks associated with high levels of stress-induced endogenous GCs or with exogenous GC treatments. Based on the clinical trials using GC administration as an adjunctive therapy for sepsis (see High- and Low-Dose GC Therapy), it seems that the main factors determining the outcome of GC therapy on the infection include the dose, duration, and timing of GC therapy.

### Effect of GR Signaling on Vascular Function

GCs also regulate different aspects of endothelial physiology during sepsis. In sepsis patients, the vascular reactivity and endothelial barrier function are impaired, and this contributes to adverse outcome (65). Possible mechanisms for reduced vascular tone include (i) induction of nitric oxide (NO) synthase, which in turn produces relaxation of vascular smooth muscle tone resulting in hypotension, and (ii) reduction of vasoconstrictor response to catecholamines.

Adrenalectomized mice show a much more severe form of circulatory shock after LPS injection, which is characterized by a profound drop in blood pressure and lack of response to intravenous noradrenaline injection (66). Adrenalectomized mice lack production of both GCs (derived from the adrenal cortex) and catecholamines (derived from the adrenal medulla). However, the synthetic GC dexamethasone is able to restore this hyporeactivity to noradrenaline injection, probably resulting from rapid non-genomic effects according to the authors as these effects are seen as early as 10 min after dexamethasone administration (67). On the other hand, this response seems to be dimer dependent, as GR^dim/dim^ mice display an aggravated hemodynamic instability after LPS challenge, reflected by a significantly increased need for norepinephrine to reach hemodynamic stability (49). GCs also enhance vasoconstrictor response to catecholamines in septic patients, and this effect was stronger in patients with adrenal insufficiency (68). Furthermore, GCs act as a negative regulator of NO release and deletion of GR in endothelial cells renders mice more susceptible for LPS-induced shock through increased hemodynamic instability (38). Repeated challenges with small doses of LPS result in tolerance to peripheral vascular hyporeactivity and associated mortality caused by subsequent injection with a higher dose of LPS. This tolerance could be explained by reduced induction of iNOS due to the elevated endogenous GC levels (69). Lastly, GR in macrophages is necessary to induce *SphK1*, which has a critical role in maintaining endothelial barrier function. Dexamethasone pretreatment protects against LPS-induced acute lung injury via maintaining the endothelial barrier integrity in the lungs (40).

### Effect of GR Signaling on Glucose Homeostasis

Disturbance in glucose homeostasis is a typical feature of sepsis. In the initial phase of sepsis, hyperglycemia is observed resulting from insulin resistance, altered glycogen metabolism, and/or increased gluconeogenesis. This allows redirection of glucose to immune cells supporting aerobic glycolysis and thus immune function. In a later stage of sepsis, hypoglycemia could be observed presumably resulting from depleted glycogen storages, increased peripheral usage of glucose, anorexia, and/or decreased gluconeogenesis (70).

The GR plays a key role in glucose homeostasis by regulating the transcription of genes coding for gluconeogenic enzymes such as *Pck1* and *G6Pc* and by reducing the glucose uptake (e.g., GC suppresses GLUT4 translocation to the membrane) and glucose utilization (e.g., GC upregulates pyruvate dehydrogenase kinase *Pdk4*) in skeletal muscle and white adipose tissue (71). Pro-inflammatory cytokines following endotoxic shock exposure has been identified to decrease expression of gluconeogenic enzymes. Both the GC-responsive element (GRE) and the cyclic adenosine monophosphate (cAMP)-responsive element (CRE) were identified as critical cis-regulatory targets of the pro-inflammatory cytokines (72). Specifically the expression of nuclear receptor cofactor peroxisome proliferator-activator receptor coactivator 1a (PGC1a), the molecular mediator of the GRE/CRE synergism on the PEPCK promoter, was found to be repressed in liver during endotoxic shock (72). Restoration of PGC1a restored PEPCK expression in hepatocytes exposed to pro-inflammatory signaling (72). *In vivo*, anti-TNF pretreatment in mice challenged with LPS is able to maintain normal *Pck1* transcription and enzyme activity, thereby identifying a role for TNF as the mediator of LPS-induced *Pck1* downregulation (73). However, neutralization of TNF fails to prevent hypoglycemia during endotoxemia (73). PEPCK was considered as the rate-limiting enzyme for a long time, but a study published by Burgess et al. surprisingly showed that hepatic PEPCK content alone is only weakly influencing gluconeogenesis (74). This suggests that other factors such as peripheral substrate supply, other gluconeogenic enzymes, and/or hepatic energy metabolism must coordinate with PEPCK expression to regulate gluconeogenesis (74). Next to PEPCK, also G6Pase is found to be downregulated after LPS (72) and CLP-induced sepsis (75). PGC1a is also known to increase the expression G6Pase, providing an extra possible mechanism in which inflammation represses gluconeogenesis and promotes hypoglycemia (76). Furthermore, ROS is able to repress G6Pase expression and pharmacological inhibition of ROS restores *G6Pc* gene expression and counteracts hypoglycemia in septic mice (75). It is thus plausible that inflammation induces hypoglycemia by affecting simultaneously multiple enzymes in the gluconeogenic pathway and GR is necessary to counteract the inflammation-induced effects.

## Dysfunction of the HPA Axis During Sepsis

### CIRCI

It is clear from the abovementioned studies that activation of the HPA axis and GC signaling are required for maintaining homeostasis and eventually survival in sepsis. However, in many critically ill patients, this homeostatic activation of adrenocortical hormones is impaired. It has been estimated that dysfunction of the HPA axis occurs in 10–20% of critically ill patients and increases to 60% in patients with septic shock (77). Adrenal necrosis and hemorrhage as a result of renal failure, shock, or disseminated intravascular coagulation (DIC) may lead to adrenal insufficiency (77). However, it seems that most patients develop reversible dysfunction of the HPA axis caused by pro-inflammatory mediators. In 2008, the term adrenal insufficiency was replaced by the term “Critical Illness-Related Corticosteroid Insufficiency” (CIRCI) (78) because adrenal insufficiency may arise due to dysfunction at any point in the HPA axis, including the hypothalamus, pituitary, and adrenal cortex. Indeed, direct damage to the hypothalamus or the pituitary gland caused by head injuries (79), the use of sedatives such as etomidate (80), long-term treatment with exogenous GCs (81), or altered metabolism of cortisol (34) may all affect the HPA-axis response in critically ill patients. Lastly, the resistance of target tissues to GCs, so-called glucocorticoid resistance (GCR), can also be interpreted as an insufficiency of the HPA axis at the peripheral level of target tissues (77). In the past, the diagnosis of adrenal insufficiency was based on the measurement of total serum cortisol or the change in the serum cortisol in response to 250 μg of ACTH (ACTH stimulation test). However, as CIRCI may result from defects at multiple levels in the HPA axis and in the periphery, the current diagnostic tools to identify patients with CIRCI turned out to be inadequate (77).

### Glucocorticoid Resistance in Sepsis

GCR is a well-known manifestation in sepsis and may contribute to the failure of GCs to improve sepsis patients. GCR refers to the inadequate response of the GR to regulate the transcription of GR-responsive genes, despite seemingly adequate plasma cortisol concentrations. Evidence for an association between the degree of GC unresponsiveness and disease severity and mortality was demonstrated in acute respiratory distress response (ARDS) (82) and septic shock (83).

**Figure 3:**
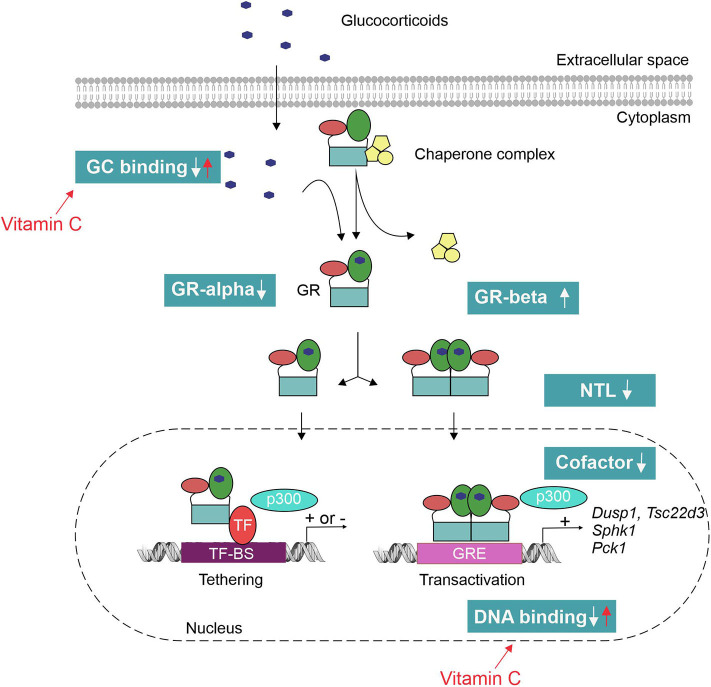
Mechanisms of glucocorticoid resistance (GCR) in sepsis. Pro-inflammatory cytokines and oxidative stress have shown to interfere with the GC signaling pathway at multiple levels. This leads to an inadequate response of the GR to both endogenous (cortisol/corticosterone) and exogenous GCs to tolerate collateral damage induced by sepsis. Vitamin C has been shown to restore the GR function by reverting the effect of oxidative stress on GR's ligand- and DNA-binding capacity. TF-BS, transcription factor binding site; GRE, GC-responsive element; NTL, nuclear translocation.

The mechanisms of GCR in sepsis, in theory, can be manifold (Figure 3). Pro-inflammatory cytokines such as TNF have been shown to decrease the GR-alpha expression (84). Reduced GR-alpha levels are reported in several cell types derived from sepsis patients (PBMC, liver, brain, muscle and lung) (85–87). However, other studies displayed an increased expression of GR-alpha upon inflammation (88, 89). Increased expression of the dominant-negative isoform GR-beta has also been shown in sepsis (90); however, increased GR-beta levels do not seem to correlate with decreased GC responses in septic shock patients (83). Next, pro-inflammatory cytokines such as IL-1 and oxidative stress may hamper nuclear translocation (NTL) of GR upon dexamethasone administration (88, 91). In an *ex vivo* model, reduced NTL of the GR in patients with lethal ARDS was demonstrated despite adequate levels of cortisol (82). This reduced NTL could possibly be explained by reduced GC-binding capacity of the GR upon endotoxin challenge (92), as GR ligand binding is necessary to expose the nuclear localization signal (NLS). A possible role for nitric oxide (NO) was found herein as prophylactic administration of a NO synthase inhibitor blunted the LPS-induced decrease in GR binding to GCs (93). Furthermore, GR binding to DNA may be affected by oxidation of cysteine thiol groups of the GR or by metal ions that have high affinity to thiol, thereby reducing the efficacy of GCs (94, 95). Finally, the GR cofactor PGC1a is found to be downregulated in septic liver (72) and cells pretreated with TNF display a reshuffling of cofactor p300 from GR to NF-κB, thereby modulating the GR interactome (96). PGC1a restoration or p300 overexpression restores GC responsiveness (72, 96).

GCR has been considered by some researchers as another good reason to treat sepsis patients with high stress doses of GCs (77), whereas other groups used GCR as an argument against the use of GCs in sepsis (34, 97). On the one hand, GCR obviously limits the therapeutic use of GCs, since there is insufficient GR functioning. In the TNF-induced SIRS model, dexamethasone only protects when given before TNF injection. However, when dexamethasone was given shortly after TNF injection, it is unable to protect (84). These results may imply that the inflammatory environment leads to reduced functioning of the GR. On the other hand, GC treatment further suppresses GR expression as was shown in human biopsy samples and this may predispose the patients to excessive inflammation, organ failure, and eventually death (34). Understanding the mechanisms underlying GCR opens avenues for reverting GCR in sepsis and applying GCs therapeutically. One of the most promising candidates to revert GCR is the antioxidant L-ascorbic acid, or vitamin C. Vitamin C has been shown to restore the GR function by reverting the effect of oxidative stress on GR's ligand- and DNA-binding capacity (98). Preclinical studies demonstrated the synergistic effect of adding vitamin C to GC therapy on endothelial barrier function (99) and intestinal mucosa injuries in sepsis models (100). Furthermore, two recent retrospective before–after clinical trials evaluating this combination therapy in the treatment of septic shock and severe pneumonia look very promising (101, 102). This combination therapy will be further discussed below. Alternatively, administration of GC-induced proteins, such as GILZ, allows working downstream of the GR and thus provides a potential mechanism to circumvent GCR in sepsis (54, 55, 103). Injection of a TAT-GILZ fusion construct into mice has been shown to protect against LPS-induced endotoxemia (31). The TAT peptide is a cell-penetrating peptide that can be used to overcome the lipophilic barrier of the cell membrane and deliver large molecules and small particles inside cells. Whether this fusion construct could circumvent GCR remains to be studied. Another way of increasing GILZ expression in cells is via administering other factors, such as vitamin D3, which is known to increase GILZ expression in DCs (104).

## A Therapeutic Role for Exogenous GCs in Sepsis?

### High- and Low-Dose GC Therapy

GCs as an adjunctive treatment for sepsis remain one of the most controversial items in sepsis despite more than four decades of investigations. The rationale for the use of GCs in sepsis is that GCs attenuate both the pro- and anti-inflammatory responses typically seen in sepsis. Besides regulating inflammation, GCs also increase the vasoactive tone, thereby amplifying the effect of vasopressors during septic shock (78). Between 1976 and 2018, 24 randomized clinical trials (RCTs) have been published reporting the 28-day mortality or hospital mortality of GC therapy in sepsis or septic shock (105). These studies exhibited conflicting results. Even the results of multiple meta-analyses were contradictory with some showing survival advantage (106, 107), whereas others did not find any survival benefit (108, 109).

The first large clinical trials on the use of GCs in sepsis involved high dose of GCs in the management of septic shock. This early study showed that bolus injection of high doses of GCs (3 mg/kg dexamethasone or 30 mg/kg methylprednisolone, equivalent to 40 g of hydrocortisone) significantly reduced mortality rates from 38.4 to 10.4% (110). Later studies however reported that short courses of high-dose GC are associated with worsened secondary infections and an increased risk of death (111).

More recent studies have evaluated the use of GCs in doses that are rather similar to supraphysiological stress doses (200–300 mg hydrocortisone/day) and over a longer time period (5–7 days) (Table 2). The recommendation of 200–300 mg of hydrocortisone as a supraphysiologic dose stems from the observation that this would be the amount of cortisol produced by a maximally stimulated adrenal gland (117). Of note, the “low dose” terminology used in sepsis is considered as high dose in for example rheumatoid arthritis (118). The rationale for the use of **low-dose GCs** in sepsis is to substitute the lack of endogenous steroid activity in phases of severe stress, instead of maximally suppressing the host immune response to infection. Indeed, during sepsis and septic shock, CIRCI leads to insufficient functioning of the HPA axis thereby contributing to the disease (77). Moreover, at “lower doses,” GCs have been shown to exert beneficial effects in terms of improvements in hemodynamics and decrease in pro-inflammatory mediators and oxidative stress without compromising bacterial resolution in patients with septic shock (78, 119).

**Table 2:** Major randomized clinical trials investigating the effect of low-dose GC therapy in sepsis or septic shock patients.

**Study**	**Year**	**# Patients**	**28-Day mortality in control group**	**Treatment start**	**Mortality benefit**	**Shock reversal**	**References**
Annane et al.	2002	299	61%	≤ 8 h	6%	2 days	(112)
CORTICUS	2008	499	36.1%	≤ 72 h	No	3.3 days	(113)
HYPRESS	2016	380	8.2%	≤ 48 h	No	No	(114)
ADRENAL	2018	3,800	24.3%	20 ± 90 h	No	1 day	(115)
APROCCHSS	2018	1,241	38.9%	≤ 24 h	6.1%	2 days	(116)

In 2002, Annane et al. showed that low doses of GCs (hydrocortisone plus fludrocortisone) significantly reduced mortality in patients with septic shock who did not respond to the ACTH stimulation test (cortisol response in serum <9 μg/dl) (112). The ACTH test was used to detect people with adrenal insufficiency. However, a second large-placebo controlled trial (CORTICUS) using the same dosage of hydrocortisone failed to show any survival benefit in either responders or non-responders to ACTH (113). Differences between these two trials comprise the lack of fludrocortisone, lower disease severity, and later entry window for patients in the CORTICUS trial. In 2016, the HYPRESS trial tested whether HYdrocortisone could preemptively PREvent the development of Septic Shock in patients with hospital-acquired sepsis, but also these results were negative (114).

In view of all these conflicting results, the 2016 Surviving Sepsis Campaign (SSC) guidelines recommend the use of GCs to treat patients with septic shock if adequate fluid resuscitation and vasopressor therapy cannot restore hemodynamic stability (120). To improve the evidence for the use of low-dose GCs in septic shock, two large-scale RCTs were conducted and results were published in *The New England Journal of Medicine* in 2018. Together, these two studies enrolled a total of 5041 patients outpacing all previous studies. The ADRENAL trial (Adjunctive Corticosteroid Treatment in Critically Ill Patients with Septic Shock) showed that treatment with hydrocortisone did not improve 90-day survival (115). The APROCCHSS trial (Activated Protein C and Corticosteroids for Human Septic Shock) in contrast showed a significant reduction in mortality rates from 49.1 to 43% (116). Both trials included patients with septic shock; however, the APROCCHSS trial enrolled patients with a more severe septic shock (90-day mortality rate in control group of 49.1 vs. 28.8% in ADRENAL). Additionally, patients in the APROCCHSS trial were enrolled earlier in the study than patients in ADRENAL trial (time from shock onset to randomization was ≤ 24 h for all patients in APROCCHSS trial vs. 20 ± 90 h in ADRENAL trial). Lastly, patients in APROCCHSS were bolus-injected with hydrocortisone combined with oral fludrocortisone in contrast to patients in ADRENAL who received a continuous infusion of hydrocortisone. Of note, both studies used a daily dose of 200 mg hydrocortisone for 7 days. Even though the two recent trials showed either no survival benefit or at best a small reduction in mortality with a low dose of GCs in septic shock, the time to resolution of shock, the time to cessation of mechanical ventilation, and the time to discharge from the ICU were significantly shorter in the GC-treated patients for both studies. Interestingly, low-dose GCs were not associated with increased risks for secondary infections, gastrointestinal bleeding, delayed wound healing, or myopathy, thereby strengthening the rationale for the use of low-dose GC therapy in sepsis.

To conclude, although GCs may have beneficial effects on the pathophysiology of septic shock, it seems only to protect in the sickest subgroup of septic shock patients when treated early after shock onset. Given the low cost and limited side effects of a 7-day GC treatment and high cost of intensive care unit stay, it is likely that future sepsis guidelines will reinforce the use of low-dose GCs for treatment of septic shock.

### Combination Therapy

There is a significant association between the impact of early initiation of GC therapy and improved survival (121, 122). Delayed initiation of GC therapy limits the therapeutic value of GCs, probably reflecting the onset of GCR after sepsis. Therefore, adding compounds that reverses GCR opens new avenues for GC therapy. As shown above, vitamin C enhances GC function by reversing the effects of oxidative stress such as oxidation of cysteine thiol groups, which affects ligand- and DNA-binding of GR (98). Inversely, the transport of vitamin C into the cell is mediated by the sodium-vitamin C transporter (SVCT2). Pro-inflammatory cytokines decrease the expression of SVCT2 (123), a process that is reversed by adding GCs (124). Additionally, vitamin C has anti-inflammatory effects (125), improves the integrity of the endothelium (126), and increases vasopressor synthesis (127). It thus seems that both GCs and vitamin C act synergistically.

As septic patients often present depletion of vitamin C, several studies evaluated the effect of high-dose vitamin C in the management of sepsis and septic shock (128, 129). High doses of vitamin C however increases oxalate production, which tends to accumulate in the kidneys of patients with renal impairment in the form of kidney stones (130). Thiamin (vitamin B1) is able to reduce this crystallization (131). Moreover, thiamin is an essential cofactor for the multi-enzyme complex pyruvate dehydrogenase (PDC), a key enzyme needed for the formation of acetyl-coA out of pyruvate to produce energy via the Krebs cycle. Thiamin as a monotherapy in septic shock patients with thiamin deficiency lowered the lactate levels at 24 h and decreased mortality rates in this subgroup of patients (132).

**Figure 4:**
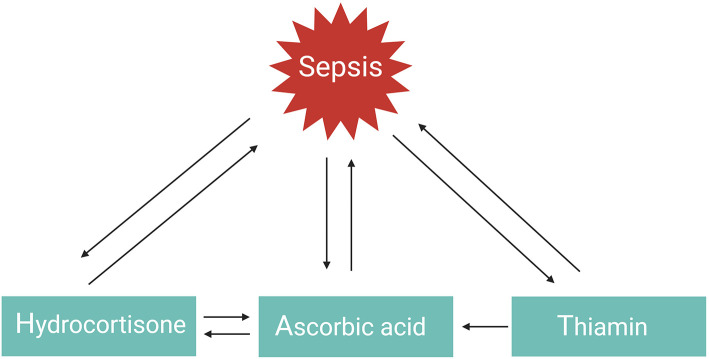
HAT therapy. The function or levels of hydrocortisone, ascorbic acid, and thiamin are reduced in sepsis. Administering one of these agents as monotherapy has shown favorable but limited outcomes. Combination of these three agents in the so-called HAT therapy may work synergistically to combat sepsis.

GCs, vitamin C, or thiamin as monotherapy have all three shown protective but limited effects in the management of septic shock. However, a combination of these three therapies in a retrospective before–after study was found to prevent progressive organ dysfunction and reduced hospital mortality from 40.4% in the control group to 8.5% in the sepsis patients receiving the combination therapy, with no increase in adverse effects (101). Similar findings were observed in patients with severe pneumonia who required admission to an ICU (hospital mortality reduced from 39 to 17%) (102). The explanation as to why the combination of hydrocortisone, vitamin C, and thiamine appeared to have such a striking effect on hospital mortality, in comparison to the myriad of studies evaluating the effect of using one single drug in previous sepsis trials, likely results from the multiple and overlapping effects of all three agents and the synergistic effect between them (Figure 4) (101). This combination therapy, also referred to as HAT therapy, is currently further being evaluated in a number of RCTs (133). Alternative combination therapies such as vitamin D3 with GCs also merit further investigation in sepsis. One study showed that vitamin D3 lowers mortality in the severe vitamin D deficiency subgroup of sepsis patients (134), but these results could not be confirmed in a recent trial (135). Interestingly, in inflammatory disorders such as psoriasis and palmoplantar pustulosis, combining GCs with vitamin D3 appears to be more effective than GC or vitamin D3 monotherapy as these two agents work synergistically (136, 137). Whether this is also the case for sepsis remains to be studied.

## Conclusion

The use of GCs as a monotherapy in sepsis remains a matter of debate. In 2018, two large-scale RCTs evaluating the effect of GCs in patients with sepsis or septic shock were published, but no definitive conclusion could be drawn. Recently, two retrospective before–after studies evaluated the efficacy of combining GC therapy with vitamin C and thiamin (HAT therapy). These studies presented very promising results, yet the sample size was rather small. Therefore, results from ongoing large randomized controlled studies are heavily awaited. Nevertheless, these preliminary, positive results renew the hope for GC therapy in sepsis and should encourage researchers to explore mechanisms that counteract GCR with the aim of reversing GCR.

## Author Contributions

JV wrote the MS. CL supervised the MS. All authors contributed to the article and approved the submitted version.

## Conflict of Interest

The authors declare that the research was conducted in the absence of any commercial or financial relationships that could be construed as a potential conflict of interest.

## References

[1] Singer M Deutschman CS Seymour C Shankar-Har M Annane D Angus DC The third international consensus definitions for sepsis and septic shock (Sepsis-3). JAMA. (2016) 315:801–10. 10.1001/jama.2016.0287 26903338 PMC4968574

[2] Reinhart K Daniels R Kissoon N Machado FR Schachter RD Finfer S. Recognizing sepsis as a global health priority—a WHO Resolution. N Engl J Med. (2017) 377:414–7. 10.1056/NEJMp1707170 28658587

[3] Rudd KE Johnson SC Agesa KM Shackelford KA Tsoi D Kievlan DR. Global, regional, and national sepsis incidence and mortality, 1990–2017: analysis for the global burden of disease study. Lancet. (2020) 395:200–11. 10.1016/S0140-6736(19)32989-7 31954465 PMC6970225

[4] Fleischmann C Scherag A Adhikari NKJ Hartog CS Tsaganos T Schlattmann P. Assessment of global incidence and mortality of hospital-treated sepsis current estimates and limitations. Am J Respir Crit Care Med. (2016) 193:259–72. 10.1164/rccm.201504-0781OC 26414292

[5] Cavaillon JM Chrétien F. From septicemia to sepsis 3.0—from Ignaz semmelweis to louis pasteur. Microbes Infect. (2019) 20:213–21. 10.1016/j.micinf.2019.06.005 31255674

[6] Arefian H Heublein S Scherag A Brunkhorst FM Younis MZ Moerer O. Hospital-related cost of sepsis: a systematic review. J Infect. (2017) 74:107–17. 10.1016/j.jinf.2016.11.006 27884733

[7] Hotchkiss RS Moldawer LL Opal SM Reinhart K Turnbull IR Vincent JL Sepsis and septic shock. Nat Rev Dis Prim. (2016) 2:16045 10.1038/nrdp.2016.45 28117397 PMC5538252

[8] Chou EH Mann S Hsu TC Hsu WT Liu CCY Bhakta T. Incidence, trends, and outcomes of infection sites among hospitalizations of sepsis: a nationwide study. PLoS ONE. (2020) 15:e0227752. 10.1371/journal.pone.0227752 31929577 PMC6957188

[9] Stortz JA Raymond SL Mira JC Moldawer LL Mohr AM Efron PA. Murine models of sepsis and trauma: can we bridge the gap? ILAR J. (2017) 58:90–105. 10.1093/ilar/ilx007 28444204 PMC5886315

[10] Dejager L Pinheiro I Dejonckheere E Libert C. Cecal ligation and puncture: the gold standard model for polymicrobial sepsis? Trends Microbiol. (2011) 19:198–208. 10.1016/j.tim.2011.01.001 21296575

[11] Denstaedt SJ Singer BH Standiford TJ. Sepsis and nosocomial infection: patient characteristics, mechanisms, and modulation. Front Immunol. (2018) 9:2446. 10.3389/fimmu.2018.02446 30459764 PMC6232897

[12] Muenzer JT Davis CG Dunne BS Unsinger J Dunne WM Hotchkiss RS. Pneumonia after cecal ligation and puncture: a clinically relevant “two-hit” model of sepsis. Shock. (2006) 26:565–70. 10.1097/01.shk.0000235130.82363.ed 17117130

[13] Restagno D Venet F Paquet C Freyburger L Allaouchiche B Monneret G. Mice survival and plasmatic cytokine secretion in a “two hit” model of sepsis depend on intratracheal pseudomonas aeruginosa bacterial load. PLoS ONE. (2016) 11:e0162109. 10.1371/journal.pone.0162109 27574993 PMC5004855

[14] Medzhitov R Schneider DS Soares MP. Disease tolerance as a defense strategy. Science. (2012) 335:936–41. 10.1126/science.1214935 22363001 PMC3564547

[15] Spiga F Walker JJ Terry JR Lightman SL. HPA axis-rhythms. Compr Physiol. (2014) 4:1273–98. 10.1002/cphy.c140003 24944037

[16] Dantzer R. Neuroimmune interactions: from the brain to the immune system and vice versa. Physiol Rev. (2018) 98:477–504. 10.1152/physrev.00039.2016 29351513 PMC5866360

[17] Walker JJ Spiga F Gupta R Zhao Z Lightman SL Terry JR. Rapid intra-adrenal feedback regulation of glucocorticoid synthesis. J R Soc Interface. (2015) 12:20140875. 10.1098/rsif.2014.0875 25392395 PMC4277077

[18] Cain DW Cidlowski JA. Immune regulation by glucocorticoids. Nat Rev Immunol. (2017) 17:233–47. 10.1038/nri.2017.1 28192415 PMC9761406

[19] Vandewalle J Luypaert A De Bosscher K Libert C. Therapeutic mechanisms of glucocorticoids. Trends Endocrinol Metab. (2018) 29:42–54. 10.1016/j.tem.2017.10.010 29162310

[20] Diamond MI Miner JN Yoshinaga SK Yamamoto KR Transcription factor interactions: selectors of positive or negative regulation from a single DNA element. Science. (1990) 249:1266–72. 10.1126/science.2119054 2119054

[21] Hudson WH Youn C Ortlund EA. The structural basis of direct glucocorticoid-mediated transrepression. Nat Struct Mol Biol. (2013) 20:53–58. 10.1038/nsmb.2456 23222642 PMC3539207

[22] Lim H Uhlenhaut NH Rauch A Weiner J Hübner S Hübner N. Genomic redistribution of GR monomers and dimers mediates transcriptional response to exogenous glucocorticoid *in vivo*. Genome Res. (2015) 25:836–44. 10.1101/gr.188581.114 25957148 PMC4448680

[23] Ratman D Vanden Berghe W Dejager L Libert C Tavernier J Beck IM. How glucocorticoid receptors modulate the activity of other transcription factors: a scope beyond tethering. Mol Cell Endocrinol. (2013) 380:41–54. 10.1016/j.mce.2012.12.014 23267834

[24] Song IH Buttgereit F. Non-genomic glucocorticoid effects to provide the basis for new drug developments. Mol Cell Endocrinol. (2006) 246:142–6. 10.1016/j.mce.2005.11.012 16388891

[25] Witek-Janusek L Yelich MR. Role of the adrenal cortex and medulla in the young rats' glucoregulatory response to endotoxin. Shock. (1995) 3:434–9. 7656068

[26] Butler LD Layman NK Riedl PE Cain RL Shellhaas J Evans GF. Neuroendocrine regulation of *in vivo* cytokine production and effects: I. *In vivo* regulatory networks involving the neuroendocrine system, interleukin-1 tumor necrosis factor-α. J Neuroimmunol. (1989) 24:143–53. 10.1016/0165-5728(89)90108-2 2681261

[27] Lazar G Lazar G Agarwal MK. Modification of septic shock in mice by the antiglucocorticoid RU 38486. Circ Shock. (1992) 36:180–4. 1611702

[28] Bancos I Hazeldine J Chortis V Hampson P Taylor AE Lord JM. Primary adrenal insufficiency is associated with impaired natural killer cell function: a potential link to increased mortality. Eur J Endocrinol. (2017) 176:471–80. 10.1530/EJE-16-0969 28223394 PMC5425935

[29] Reichardt HM Umland T Bauer A Kretz O Schutz G. Mice with an increased glucocorticoid receptor gene dosage show enhanced resistance to stress and endotoxic shock. Mol Cell Biol. (2000) 20:9009–17. 10.1128/MCB.20.23.9009-9017.2000 11073999 PMC86554

[30] Dejager L Pinheiro I Puimège L Fan YD Gremeaux L Vankelecom H. Increased glucocorticoid receptor expression and activity mediate the LPS resistance of SPRET/EI mice. J Biol Chem. (2010) 285:31073–86. 10.1074/jbc.M110.154484 20663891 PMC2945598

[31] Pinheiro I Dejager L Petta I Vandevyver S Puimege L Mahieu T. LPS resistance of SPRET/Ei mice is mediated by Gilz, encoded by the Tsc22d3 gene on the X chromosome. EMBO Mol Med. (2013) 5:456–70. 10.1002/emmm.201201683 23495141 PMC3598084

[32] Vandermosten L Vanhorebeek I De Bosscher K Opdenakker G Van den Steen PE. Critical roles of endogenous glucocorticoids for disease tolerance in malaria. Trends Parasitol. (2019) 35:918–30. 10.1016/j.pt.2019.08.007 31606404

[33] Weikum ER Knuesel MT Ortlund EA Yamamoto KR. Glucocorticoid receptor control of transcription: precision and plasticity via allostery. Nat Rev Mol Cell Biol. (2017) 18:159–74. 10.1038/nrm.2016.152 28053348 PMC6257982

[34] Jenniskens M Weckx R Dufour T Vander Perre S Pauwels L Derde S. The hepatic glucocorticoid receptor is crucial for cortisol homeostasis and sepsis survival in humans and male mice. Endocrinology. (2018) 159:2790–802. 10.1210/en.2018-00344 29788135

[35] Ballegeer M Van Looveren K Timmermans S Eggermont M Vandevyver S Thery F. Glucocorticoid receptor dimers control intestinal STAT1 and TNF-induced inflammation in mice. J Clin Invest. (2018) 128:3265–79. 10.1172/JCI96636 29746256 PMC6063488

[36] Van Looveren K Timmermans S Vanderhaeghen T Wallaeys C Ballegeer M Souffriau J Glucocorticoids limit lipopolysaccharide-induced lethal inflammation by a double control system. EMBO Rep. (2020) 8:e49762 10.15252/embr.201949762 PMC7332980 32383538

[37] Braun TP Grossberg AJ Krasnow SM Levasseur PR Szumowski M Zhu XX. Cancer- and endotoxin-induced cachexia require intact glucocorticoid signaling in skeletal muscle. FASEB J. (2013) 27:3572–82. 10.1096/fj.13-230375 23733748 PMC3752537

[38] Goodwin JE Feng Y Velazquez H Sessa WC. Endothelial glucocorticoid receptor is required for protection against sepsis. Proc Natl Acad Sci USA. (2013) 110:306–11. 10.1073/pnas.1210200110 23248291 PMC3538225

[39] Bhattacharyya S Brown DE Brewer JA Vogt SK Muglia LJ Macrophage glucocorticoid receptors regulate Toll-like receptor 4–mediated in ammatory responses by selective inhibition of p38 MAP kinase. Blood. (2007) 109:4313–19. 10.1182/blood-2006-10-048215 17255352 PMC1885507

[40] Vettorazzi S Bode C Dejager L Frappart L Shelest E Klaßen C. Glucocorticoids limit acute lung inflammation in concert with inflammatory stimuli by induction of SphK1. Nat Commun. (2015) 6:7796. 10.1038/ncomms8796 26183376 PMC4518295

[41] Kleiman A Hubner S Rodriguez Parkitna JM Neumann A Hofer S Weigand MA. Glucocorticoid receptor dimerization is required for survival in septic shock via suppression of interleukin-1 in macrophages. FASEB J. (2012) 26:722–9. 10.1096/fj.11-192112 22042221

[42] Guo L Zheng Z Ai J Howatt DA Mittelstadt PR Thacker S. Scavenger receptor BI and high-density lipoprotein regulate thymocyte apoptosis in sepsis. Arterioscler Thromb Vasc Biol. (2014) 34:966–75. 10.1161/ATVBAHA.113.302484 24603680 PMC4010389

[43] Li CC Munitic I Mittelstadt PR Castro E Ashwell JD. Suppression of dendritic cell-derived IL-12 by endogenous glucocorticoids is protective in LPS-induced sepsis. PLoS Biol. (2015) 13:e1002269. 10.1371/journal.pbio.1002269 26440998 PMC4595142

[44] Quatrini L Wieduwild E Escaliere B Filtjens J Chasson L Laprie C. Endogenous glucocorticoids control host resistance to viral infection through the tissue-specific regulation of PD-1 expression on NK cells. Nat Immunol. (2018) 19:954–62. 10.1038/s41590-018-0185-0 30127438 PMC6138242

[45] Quatrini L Wieduwild E Guia S Bernat C Glaichenhaus N Vivier E. Host resistance to endotoxic shock requires the neuroendocrine regulation of group 1 innate lymphoid cells. J Exp Med. (2017) 214:3531–41. 10.1084/jem.20171048 29141867 PMC5716043

[46] King EM Chivers JE Rider CF Minnich A Giembycz MA Newton R. Glucocorticoid repression of inflammatory gene expression shows differential responsiveness by transactivation- and transrepression-dependent mechanisms. PLoS ONE. (2013) 8:e53936. 10.1371/journal.pone.0053936 23349769 PMC3545719

[47] Reichardt HM Kaestner KH Tuckermann J Kretz O Wessely O Bock R DNA binding of the glucocorticoid receptor is not essential for survival. Cell. (1998) 93:531–41. 10.1016/S0092-8674(00)81183-6 9604929

[48] Vandevyver S Dejager L Van Bogaert T Kleyman A Liu Y Tuckermann J. Glucocorticoid receptor dimerization induces MKP1 to protect against TNF-induced inflammation. J Clin Invest. (2012) 122:2130–40. 10.1172/JCI60006 22585571 PMC3366401

[49] Wepler M Preuss JM Merz T Hartmann C Wachter U McCook O. (2020) Impaired glucocorticoid receptor dimerization aggravates LPS-induced circulatory and pulmonary dysfunction. Front. Immunol. 10:3152. 10.3389/fimmu.2019.03152 32038649 PMC6990631

[50] Thomsen KL Møller HJ Graversen JH Magnusson NE Moestrup SK Vilstrup H. Anti-CD163-dexamethasone conjugate inhibits the acute phase response to lipopolysaccharide in rats. World J Hepatol. (2016) 8:726–30. 10.4254/wjh.v8.i17.726 27330681 PMC4911506

[51] Van Der Goes A Hoekstra K Van Den Berg TK Dijkstra CD. Dexamethasone promotes phagocytosis and bacterial killing by human monocytes / macrophages *in vitro*. J Leukoc Biol. (2000) 67:801–7. 10.1002/jlb.67.6.801 10857852

[52] Keh D Boehnke T Weber-Cartens S Schulz C Ahlers O Bercker S. Immunologic and hemodynamic effects of “low-dose” hydrocortisone in septic shock: a double-blind, randomized, placebo-controlled, crossover study. Am J Respir Crit Care Med. (2003) 167:512–20. 10.1164/rccm.200205-446OC 12426230

[53] Doulias T Quickert S Weis S Claus RA Kontopoulou K Giamarellos-Bourboulis EJ. Low-dose hydrocortisone prolongs survival in a lethal sepsis model in adrenalectomized rats. J Surg Res. (2018) 227:72–80. 10.1016/j.jss.2018.02.011 29804866

[54] Ballegeer M Vandewalle J Eggermont M Van Isterdael G Dejager L De Bus L. Overexpression of gilz protects mice against lethal septic peritonitis. Shock. (2019) 52:208–14. 10.1097/SHK.0000000000001252 30124596

[55] Ellouze M Vigouroux L Tcherakian C Woerther PL Guguin A Robert O. Overexpression of GILZ in macrophages limits systemic inflammation while increasing bacterial clearance in sepsis in mice. Eur J Immunol. (2019) 50:589–602. 10.1002/eji.201948278 31840802

[56] Desgeorges T Caratti G Mounier R Tuckermann J Chazaud B. Glucocorticoids shape macrophage phenotype for tissue repair. Front Immunol. (2019) 10:1591. 10.3389/fimmu.2019.01591 31354730 PMC6632423

[57] Ehrchen J Steinmüller L Barczyk K Tenbrock K Nacken W Eisenacher M. Glucocorticoids induce differentiation of a specifically activated, anti-inflammatory subtype of human monocytes. Blood. (2007) 109:1265–74. 10.1182/blood-2006-02-001115 17018861

[58] Mittelstadt PR Monteiro JP Ashwell JD. Thymocyte responsiveness to endogenous glucocorticoids is required for immunological fitness. J Clin Invest. (2012) 122:2384–94. 10.1172/JCI63067 22653054 PMC3386828

[59] Shimba A Cui G Tani-ichi S Ogawa M Abe S Okazaki F. Glucocorticoids drive diurnal oscillations in T cell distribution and responses by inducing interleukin-7 receptor and CXCR4. Immunity. (2018) 48:286–98. 10.1016/j.immuni.2018.01.004 29396162

[60] Vétillard M Schlecht-Louf G. Glucocorticoid-induced leucine zipper: fine-tuning of dendritic cells function. Front Immunol. (2018) 9:1232. 10.3389/fimmu.2018.01232 29915587 PMC5994841

[61] Olivares-Morales MJ De La Fuente MK Dubois-Camacho K Parada D Diaz-Jiménez D Torres-Riquelme A. Glucocorticoids impair phagocytosis and inflammatory response against crohn's disease-associated adherent-invasive *Escherichia coli*. Front Immunol. (2018) 9:1026. 10.3389/fimmu.2018.01026 29867993 PMC5964128

[62] Hicks CW Sweeney DA Danner RL Eichacker PQ Suffredini AF Feng J. Beneficial effects of stress-dose corticosteroid therapy in canines depend on the severity of staphylococcal pneumonia. Intensive Care Med. (2012) 38:2063–71. 10.1007/s00134-012-2735-5 23111805 PMC4184268

[63] Stolberg VR McCubbrey AL Freeman CM Brown JP Crudgington SW Taitano SH. Glucocorticoid-augmented efferocytosis inhibits pulmonary pneumococcal clearance in mice by reducing alveolar macrophage bactericidal function. J Immunol. (2015) 195:174–84. 10.4049/jimmunol.1402217 25987742 PMC4475455

[64] Miyata M Lee JY Susuki-Miyata S Wang WY Xu H Kai H. Glucocorticoids suppress inflammation via the upregulation of negative regulator IRAK-M. Nat Commun. (2015) 6:6062. 10.1038/ncomms7062 25585690 PMC4309435

[65] Crouser ED Matthay MA. Endothelial damage during septic shock: significance and implications for future therapies. Chest. (2017) 152:1–3. 10.1016/j.chest.2017.02.016 28693760

[66] Szabo C Thiemermann C Vane JR. Inhibition of the production of nitric oxide and vasodilator prostaglandins attenuates the cardiovascular response to bacterial endotoxin in adrenalectomized rats. Proc R Soc B Biol Sci. (1993) 253:233–8. 7694300 10.1098/rspb.1993.0108

[67] Shi WL Zhang T Zhou JR Huang YH Jiang CL. Rapid permissive action of dexamethasone on the regulation of blood pressure in a rat model of septic shock. Biomed Pharmacother. (2016) 84:1119–25. 10.1016/j.biopha.2016.10.029 27780141

[68] Annane D Bellissant E Sebille V Lesieur O Mathieu B Raphael JC. Impaired pressor sensitivity to noradrenaline in septic shock patients with and without impaired adrenal function reserve Br J Clin Pharmacol. (1998) 46:589–97. 9862249 10.1046/j.1365-2125.1998.00833.x PMC1873798

[69] Szabó C Thiemermann C Wu CC Perretti M Vane JR. Attenuation of the induction of nitric oxide synthase by endogenous glucocorticoids accounts for endotoxin tolerance in vivo Proc Natl Acad Sci USA. (1994) 91:271–5. 10.1073/pnas.91.1.271 7506416 PMC42929

[70] Van Wyngene L Vandewalle J Libert C. Reprogramming of basic metabolic pathways in microbial sepsis: therapeutic targets at last? EMBO Mol Med. (2018) 10:e8712. 10.15252/emmm.201708712 29976786 PMC6079534

[71] Kuo T McQueen A Chen TC Wang JC. Regulation of glucose homeostasis by glucocorticoids. Adv Exp Med Biol. (2015) 872:99–126. 10.1007/978-1-4939-2895-8_5 26215992 PMC6185996

[72] Chichelnitskiy E Vegiopoulos A Diaz MB Ziegler A Kleiman A Rauch A. *In vivo* phosphoenolpyruvate carboxykinase promoter mapping identifies disrupted hormonal synergism as a target of inflammation during sepsis in mice. Hepatology. (2009) 50:1963–71. 10.1002/hep.23194 19821526

[73] Hill MR McCallum RE. Identification of tumor necrosis factor as a transcriptional regulator of the phosphoenolpyruvate carboxykinase gene following endotoxin treatment of mice. Infect Immun. (1992) 60:4040–50. 10.1128/IAI.60.10.4040-4050.1992 1398916 PMC257434

[74] Burgess SC He TT Yan Z Lindner J Sherry AD Malloy CR Cytosolic phosphoenolpyruvate carboxykinase does not solely control the rate of hepatic gluconeogenesis in the intact mouse liver. Cell Metab. (2007) 5:313–20. 10.1016/j.cmet.2007.03.004 17403375 PMC2680089

[75] Weis S Carlos AR Moita MR Singh S Blankenhaus B Cardoso S. Metabolic adaptation establishes disease tolerance to sepsis. Cell. (2017) 169:1263–75.e14. 10.1016/j.cell.2017.05.031 28622511 PMC5480394

[76] Rhee J Inoue Y Yoon JC Puigserver P Fan M Gonzalez FJ. Regulation of hepatic fasting response by PPARγ coactivator-1α (PGC-1): Requirement for hepatocyte nuclear factor 4α in gluconeogenesis. Proc Natl Acad Sci USA. (2003) 100:4012–7. 10.1073/pnas.0730870100 12651943 PMC153039

[77] Annane D Pastores SM Arlt W Balk RA Beishuizen A Briegel J Critical illness-related corticosteroid insufficiency (CIRCI): a narrative review from a multispecialty task force of the society of critical care medicine (SCCM) and the European society of intensive care medicine (ESICM). Intensive Care Med. (2017) 43:1781–92. 10.1007/s00134-017-4914-x 28940017

[78] Marik PE Pastores SM Annane D Meduri GU Sprung CL Arlt W. Recommendations for the diagnosis and management of corticosteroid insufficiency in critically ill adult patients: consensus statements from an international task force by the American college of critical care medicine. Crit Care Med. (2008) 36:1937–49. 10.1097/CCM.0b013e31817603ba 18496365

[79] Krahulik DD Zapletalova J Frysak Z Vaverka M. Dysfunction of hypothalamic-hypophysial axis after traumatic brain injury in adults: clinical article J Neurosurg. (2010) 113:581–4. 10.3171/2009.10.JNS09930 19929195

[80] Bruder EA Ball IM Ridi S Pickett W Hohl C. Single induction dose of etomidate versus other induction agents for endotracheal intubation in critically ill patients. Cochrane Database Syst Rev. (2015) 1:CD010225. 10.1002/14651858.CD010225.pub2 25568981 PMC6517008

[81] McDonough AK Curtis JR Saag KG. The epidemiology of glucocorticoid-associated adverse events. Curr Opin Rheumatol. (2008) 20:131–7. 10.1097/BOR.0b013e3282f51031 18349741

[82] Meduri GU Muthiah MP Carratu P Eltorky M Chrousos GP. Nuclear factor-κB- and glucocorticoid receptor α-mediated mechanisms in the regulation of systemic and pulmonary inflammation during sepsis and acute respiratory distress syndrome: evidence for inflammation-induced target tissue resistance to glucocortico. Neuroimmunomodulation. (2005) 12:321–38. 10.1159/000091126 16557033

[83] Cohen J Pretorius CJ Ungerer JPJ Cardinal J Blumenthal A Presneill J. Glucocorticoid sensitivity is highly variable in critically ill patients with septic shock and is associated with disease severity. Crit Care Med. (2016) 44:1034–41. 10.1097/CCM.0000000000001633 26963327

[84] Van Bogaert T Vandevyver S Dejager L Van Hauwermeiren F Pinheiro I Petta I Tumor necrosis factor inhibits glucocorticoid receptor function in mice: a strong signal toward lethal shock. J Biol Chem. (2011) 286:26555–67. 10.1074/jbc.M110.212365 21646349 PMC3143620

[85] Van Den Akker ELT Koper JW Joosten K De Jong FH Hazelzet JA Lamberts SWJ. Glucocorticoid receptor mRNA levels are selectively decreased in neutrophils of children with sepsis. Intensive Care Med. (2009) 35:1247–54. 10.1007/s00134-009-1468-6 19373457 PMC2698978

[86] Dekelbab BH Witchel SF DeFranco DB. TNF-α and glucocorticoid receptor interaction in L6 muscle cells: a cooperative downregulation of myosin heavy chain. Steroids. (2007) 72:705–12. 10.1016/j.steroids.2007.05.007 17624386 PMC2525668

[87] Bergquist M Nurkkala M Rylander C Kristiansson E Hedenstierna G Lindholm C. Expression of the glucocorticoid receptor is decreased in experimental staphylococcus aureus sepsis. J Infect. (2013) 67:574–83. 10.1016/j.jinf.2013.07.028 23933016

[88] Pariante CM Pearce BD Pisell TL Sanchez CI Po C Su C. The proinflammatory cytokine, interleukin-1α, reduces glucocorticoid receptor translocation and function. Endocrinology. (1999) 140:4359–66. 10.1210/endo.140.9.6986 10465310

[89] Vardas K Ilia S Sertedaki A Charmandari E Briassouli E Goukos D. Increased glucocorticoid receptor expression in sepsis is related to heat shock proteins, cytokines, and cortisol and is associated with increased mortality. Intensive Care Med Exp. (2017) 5:10. 10.1186/s40635-017-0123-8 28224564 PMC5319939

[90] Guerrero J Gatica HA Rodríguez M Estay R Goecke IA. Septic serum induces glucocorticoid resistance and modifies the expression of glucocorticoid isoforms receptors: a prospective cohort study and *in vitro* experimental assay. Crit Care. (2013) 17:R107. 10.1186/cc12774 23759144 PMC4056039

[91] Okamoto K Tanaka H Ogawa H Makino Y Eguchi H Hayashi SI. Redox-dependent regulation of nuclear import of the glucocorticoid receptor. J Biol Chem. (1999) 274:10363–71. 10.1016/S0928-4680(98)80643-3 10187825

[92] Bergquist M Jirholt P Nurkkala M Rylander C Hedenstierna G Lindholm C Glucocorticoid receptor function is decreased in neutrophils during endotoxic shock. J Infect. (2014) 6:113–22. 10.1016/j.jinf.2014.03.011 24657243

[93] Duma D Silva-Santos JE Assreuy J. Inhibition of glucocorticoid receptor binding by nitric oxide in endotoxemic rats. Crit Care Med. (2004) 32:2304–10. 10.1097/01.CCM.0000145996.57901.D7 15640646

[94] Bodwell JE Holbrook NJ Munck A. Sulfhydryl-modifying reagents reversibly inhibit binding of glucocorticoid-receptor complexes to DNA-cellulose. Biochemistry. (1984) 23:1392–8. 10.1021/bi00302a009 6722099

[95] Makino Y Tanaka H Dahlman-Wright K Makino I. Modulation of glucocorticoid-inducible gene expression by metal ions. Mol Pharmacol. (1996) 49:612–20. 8609888

[96] Dendoncker K Timmermans S Vandewalle J Eggermont M Lempiäinen J Van Hamme E. TNF-α inhibits glucocorticoid receptor-induced gene expression by reshaping the GR nuclear cofactor profile. Proc Natl Acad Sci USA. (2019) 116:12942–51. 10.1073/pnas.1821565116 31182584 PMC6600915

[97] Téblick A Peeters B Langouche L Van den Berghe G. Adrenal function and dysfunction in critically ill patients. Nat Rev Endocrinol. (2019) 15:417–27. 10.1038/s41574-019-0185-7 30850749

[98] Okamoto K Tanaka H Makino Y Makino I Restoration of the glucocorticoid receptor function by the phosphodiester compound of vitamins C and E, EPC-K1 L-ascorbic acid 2-[3,4-dihydro-2,5,7,8-tetramethyl-2-(4,8,12-trimethyltridecyl)-2H-1-benzopyran-6- yl hydrogen phosphate] potassium salt), via a Redox-Dependent Mechanism. Biochem Pharmacol. (1998) 56:79–86. 9698091 10.1016/s0006-2952(98)00121-x

[99] Barabutis N Khangoora V Marik PE Catravas JD. Hydrocortisone and ascorbic acid synergistically prevent and repair lipopolysaccharide-induced pulmonary endothelial barrier dysfunction. Chest. (2017) 152:954–62. 10.1016/j.chest.2017.07.014 28739448 PMC5812759

[100] Tavasoli M Azari O Kheirandish R Abbasi MF Evaluation of combination therapy with hydrocortisone, vitamin C, and vitamin E in a rat model of intestine ischemia-reperfusion injury. Comp Clin Path. (2018) 27: 443–39. 10.1007/s00580-017-2610-4

[101] Marik PE Khangoora V Rivera R Hooper MH Catravas J. Hydrocortisone, Vitamin C, and thiamine for the treatment of severe sepsis and septic shock: a retrospective before-after study. Chest. (2017) 151:1229–38. 10.1016/j.chest.2016.11.036 27940189

[102] Kim WY Jo EJ Eom JS Mok J Kim MH Kim KU. Combined vitamin C, hydrocortisone, and thiamine therapy for patients with severe pneumonia who were admitted to the intensive care unit: propensity score-based analysis of a before-after cohort study. J Crit Care. (2018) 47:211–8. 10.1016/j.jcrc.2018.07.004 30029205

[103] Vandewalle J Libert C. GILZ in sepsis: “Poor is the pupil who does not surpass his master,” Eur J Immunol. (2020) 50:490–3. 10.1002/eji.202048582 32103492

[104] Zimmer A Bouley J Le Mignon M Pliquet E Horiot S Turfkruyer M. A regulatory dendritic cell signature correlates with the clinical efficacy of allergen-specific sublingual immunotherapy. J Allergy Clin Immunol. (2012) 129:1020–30. 10.1016/j.jaci.2012.02.014 22464673

[105] Rygård SL Butler E Granholm A Møller MH Cohen J Finfer S. Low-dose corticosteroids for adult patients with septic shock: a systematic review with meta-analysis and trial sequential analysis. Intensive Care Med. (2018) 44:1003–16. 10.1007/s00134-018-5197-6 29761216

[106] Annane D Bellissant E Bollaert PE Briegel J Keh D Kupfer Y. Corticosteroids for treating sepsis. Cochrane Database Syst Rev. (2015) 12:CD002243. 10.1002/14651858.CD002243.pub3 26633262 PMC6494587

[107] Annane D Bellissant E Bollaert PE Briegel J Confalonieri M De Gaudio R. Corticosteroids in the treatment of severe sepsis and septic shock in adults: a systematic review. JAMA. (2009) 301:2362–75. 10.1001/jama.2009.815 19509383

[108] Sligl WI Milner DA Jr Sundar S Mphatswe W Majumdar SR. Safety and efficacy of corticosteroids for the treatment of septic shock: a systematic review and meta-analysis. Clin Infect Dis. (2009) 49:93–101. 10.1086/599343 19489712

[109] Volbeda M Wetterslev J Gluud C Zijlstra JG van der Horst ICC Keus F. Glucocorticosteroids for sepsis: systematic review with meta-analysis and trial sequential analysis. Intensive Care Med. (2015) 41:1220–34. 10.1007/s00134-015-3899-6 26100123 PMC4483251

[110] Schumer W. Steroids in the treatment of clinical septic shock. Ann Surg. (1976) 184: 333–41. 10.1097/00000658-197609000-00011 786190 PMC1344393

[111] Minneci PC Deans KJ Eichacker PQ Natanson C. The effects of steroids during sepsis depend on dose and severity of illness: an updated meta-analysis. Clin Microbiol Infect. (2009) 15:308–18. 10.1111/j.1469-0691.2009.02752.x 19416302 PMC3383780

[112] Annane D Sébille V Charpentier C Bollaert PE François B Korach JM. Effect of treatment with low doses of hydrocortisone and fludrocortisone on mortality in patients with septic shock. J Am Med Assoc. (2002) 288:862–71. 10.1001/jama.288.7.862 12186604

[113] Sprung CL Annane D Keh D Moreno R Singer M Freivogel K. Hydrocortisone therapy for patients with septic shock. N Engl J Med. (2008) 358:111–24. 10.1056/NEJMoa071366 18184957

[114] Keh D Trips E Marx G Wirtz SP Abduljawwad E Bercker S. Effect of hydrocortisone on development of shock among patients with severe sepsis the HYPRESS randomized clinical trial. JAMA. (2016) 316:1775–785. 10.1001/jama.2016.14799 27695824

[115] Venkatesh B Finfer S Cohen J Rajbhandari D Arabi Y Bellomo R. Adjunctive glucocorticoid therapy in patients with septic shock. N Engl J Med. (2018) 378:797–808. 10.1056/NEJMoa1705835 29347874

[116] Annane D Renault A Brun-Buisson C Megarbane B Quenot J-P Siami S. Hydrocortisone plus fludrocortisone for adults with septic shock. N Engl J Med. (2018) 378:809–18. 10.1056/NEJMoa1705716 29490185

[117] de Lange DW Kars M Perioperative glucocorticosteroid supplementation is not supported by evidence. Eur J Intern Med. (2008) 19:461–7. 10.1016/j.ejim.2007.12.004 18848181

[118] Buttgereit F Da Silva JPA Boers M Burmester GR Cutolo M Jacobs J. Standardised nomenclature for glucocorticoid dosages and glucocorticoid treatment regimens: current questions and tentative answers in rheumatology. Ann Rheum Dis. (2002) 61:718–22. 10.1136/ard.61.8.718 12117678 PMC1754188

[119] Kaufmann I Briegel J Schliephake F Hoelzl A Chouker A Hummel T. Stress doses of hydrocortisone in septic shock: beneficial effects on opsonization-dependent neutrophil functions. Intensive Care Med. (2008) 34:344–9. 10.1007/s00134-007-0868-8 17906853

[120] Rhodes A Evans LE Alhazzani W Levy MM Antonelli M Ferrer R. Surviving sepsis campaign: international guidelines for management of sepsis and septic shock: 2016. Intensive Care Med. (2017) 43:304–77. 10.1007/s00134-017-4683-6 28101605

[121] Park HY Suh GY Song JU Yoo H Jo IJ Shin TG. Early initiation of low-dose corticosteroid therapy in the management of septic shock: a retrospective observational study. Crit Care. (2012) 16:R3. 10.1186/cc10601 22226237 PMC3396228

[122] Katsenos CS Antonopoulou AN Apostolidou EN Ioakeimidou A Kalpakou GT Papanikolaou MN. Early administration of hydrocortisone replacement after the advent of septic shock: impact on survival and immune response. Crit Care Med. (2014) 42:1651–7. 10.1097/CCM.0000000000000318 24674923

[123] Seno T Inoue N Matsui K Ejiri J Hirata KI Kawashima S. Functional expression of sodium-dependent vitamin C transporter 2 in human endothelial cells. J Vasc Res. (2004) 41:345–51. 10.1159/000080525 15340249

[124] Fujita I Hirano J Itoh N Nakanishi T Tanaka K. Dexamethasone induces sodium-dependant vitamin C transporter in a mouse osteoblastic cell line MC3T3-E1. Br J Nutr. (2001) 86:145–9. 10.1079/BJN2001406 11502226

[125] Wannamethee SG Lowe GDO Rumley A Bruckdorfer KR Whincup PH. Associations of vitamin C status, fruit and vegetable intakes, and markers of inflammation and hemostasis. Am J Clin Nutr. (2006) 83:567–74. 10.1093/ajcn.83.3.567 16522902

[126] May JM Harrison FE. Role of vitamin C in the function of the vascular endothelium. Antioxidants Redox Signal. (2013) 19:2068–83. 10.1089/ars.2013.5205 23581713 PMC3869438

[127] Carr AC Shaw GM Fowler AA Natarajan R. Ascorbate-dependent vasopressor synthesis: a rationale for vitamin C administration in severe sepsis and septic shock? Crit Care. (2015) 19:418. 10.1186/s13054-015-1131-2 26612352 PMC4661979

[128] Zabet M Mohammadi M Ramezani M Khalili H. Effect of high-dose Ascorbic acid on vasopressor′s requirement in septic shock. J Res Pharm Pract. (2016) 5:94–100. 10.4103/2279-042X.179569 27162802 PMC4843590

[129] Fowler AA Syed AA Knowlson S Sculthorpe R Farthing D DeWilde C. Phase I safety trial of intravenous ascorbic acid in patients with severe sepsis. J Transl Med. (2014) 12:32. 10.1186/1479-5876-12-32 24484547 PMC3937164

[130] Massey LK Liebman M Kynast-Gales SA. Ascorbate increases human oxaluria and kidney stone risk. J Nutr. (2005) 135:1673–7. 10.1093/jn/135.7.1673 15987848

[131] Hoppe B Beck BB Milliner DS. The primary hyperoxalurias *Kidney Int*. (2009) 75:1264–71. 10.1038/ki.2009.32 19225556 PMC4577278

[132] Donnino MW Andersen LW Chase M Berg KM Tidswell M Giberson T. Randomized, double-blind, placebo-controlled trial of thiamine as a metabolic resuscitator in septic shock: a pilot study. Crit Care Med. (2016) 44:360–7. 10.1097/CCM.0000000000001572 26771781 PMC4754670

[133] Hager DN Hooper MH Bernard GR Busse LW Ely EW Fowler AA. The Vitamin C, thiamine and steroids in sepsis (VICTAS) protocol: a prospective, multi-center, double-blind, adaptive sample size, randomized, placebo-controlled, clinical trial. Trials. (2019) 20:197. 10.1186/s13063-019-3254-2 30953543 PMC6451231

[134] Amrein K Schnedl C Holl A Riedl R Christopher KB Pachler C Effect of high-dose vitamin D3on hospital length of stay in critically ill patients with vitamin D deficiency: the VITdAL-ICU randomized clinical trial. JAMA. (2014) 312:1520–30. 10.1001/jama.2014.13204 25268295

[135] Ginde AA Brower RG Caterino JM Finck L Banner-Goodspeed VM Grissom CK. Early high-dose Vitamin D3 for critically ill, Vitamin D–deficient patients. N Engl J Med. (2019) 381:2529–40. 10.1056/NEJMoa1911124 31826336 PMC7306117

[136] Fenton C Plosker GL. Calcipotriol/betamethasone dipropionate: a review of its use in the treatment of psoriasis vulgaris. Am J Clin Dermatol. (2004) 5:463–78. 10.2165/00128071-200405060-00012 15663344

[137] Muro M Kawakami H Matsumoto Y Abe N Tsuboi R Okubo Y. Topical combination therapy with Vitamin D3 and corticosteroid ointment for palmoplantar pustulosis: a prospective, randomized, left-right comparison study. J Dermatolog Treat. (2016) 27:51–3. 10.3109/09546634.2015.1052036 26108445

